# Prefrontal Cortex Control of Walking: Functional Near-Infrared Spectroscopy and Beyond

**DOI:** 10.1093/geroni/igaa057.2867

**Published:** 2020-12-16

**Authors:** Andrea Rosso, Roee Holtzer

## Abstract

Cognitive control of walking may change with aging and is associated with poorer mobility and greater fall risk. The prefrontal cortex function is important for cognitive control of walking, and functional near-infrared spectroscopy (fNIRS) provides the primary means for assessing prefrontal activation during walking. Growing interest in fNIRS to assess cognitive control of walking has led to advancements in the methodologies for processing and analyzing the data, a greater sophistication of experimental protocols and participant samples, and implementation within intervention studies. These advancements will be highlighted in five presentations from an international group of researchers at the forefront of the field. First, Meltem Izzetoglu will provide direct comparisons of various data processing methodologies, demonstrating comparability across approaches. Three talks will demonstrate the range of applications of fNIRS to studying walking in older adults. Nemin Chen will present data on task-related patterns of prefrontal activation across walking tasks in relation to performance, cognitive function, and structural brain health. Sarah Fraser will present results from stair climbing, a critical task for daily function which also presents a fall risk. Inbal Maidan will examine how individual differences affect prefrontal activity during walking across older adults, younger adults, and patients with Parkinson’s disease or multiple sclerosis. Finally, David Clark will demonstrate the use of fNIRS in assessing outcomes from an intervention that combined walking with non-invasive frontal brain stimulation. Roee Holtzer will lead a discussion of the results and the future of fNIRS in assessing cognitive control of walking in older adults.

